# Our New York Letter

**Published:** 1890-12

**Authors:** 


### OUR NEW YORK LETTER.

New York, November 19, 1890.

Dr. Charles McBurney, professor of surgery at the College of Physicians and Surgeons, of this city, has adopted a modification of Theirsch's method of skin-grafting in his operations at Roosevelt Hospital. He thinks that this procedure is destined soon to displace all the older methods, many of which are in constant use to-day. The length of time in which healing takes place after the operation varies from one week to two months, and some of the best results have been secured in cases where there was extensive loss of tissue. He considers the application of this method will be more extensive than it is at present, and that any one who gives it a fair trial cannot but be convinced of its great practical utility. Its applicability covers a wide range of usefulness, including ulcerated surfaces in any part of the body, varicose ulcers, burns, sloughing processes, etc.

It is remarkable, he says, to see the great variety of tissue to which these grafts will adhere, muscle, fascia, cartilage and even bone, the most favorable being muscular tissue, and the least favorable bone. At a recent clinic at Roosevelt Hospital, after the removal of a large carcinoma from the face of a patient, a considerable area of the lower jaw was denuded of tissue. Grafts were applied over the denuded surface, and at the end of a week they were found closely adhered to the bone throughout its entire extent.

Theirsch, in his transplantations, avoids all strong antiseptics, as their effect upon the tissues is to prevent immediate union of the grafts and the surface upon which they are placed. He therefore employs a solution of common salt in water in the proportion of six parts of the former to one thousand of the latter. In Roosevelt Hospital this water is sterilized and the solution boiled before using. Dr. McBurney first disinfects the ulcer with a solution of bichloride of mercury, as well as the surface from which the grafts are to be taken. He then washes away this solution with the saline one of Thiersch before operating.

The question of hemorrhage he considers a matter of considerable moment. If the grafts are placed in position before the hemorrhage has been arrested, the risk of failure is much increased, for accumulation of blood clots beneath the grafts, he says, induces necrosis, and saturation of the dressing with blood is favorable for infection. He has found that the use of an Esmarck bandage in these cases does away with all danger of hemorrhage, and materially shortens the entire operation.

The grafts are usually derived from the patient himself, the most favorable parts being the anterior and outer aspects of the thigh and outer surface of the upper arm. The grafts are excised with a razor, which is moistened with the saline solution. When they have been placed on the denuded surface, a few drops of the salt solution may be sprinkled on them from time to time so that they may not become too dry, as moisture is absolutely essential to the vitality of the transplanted skin. Strips of thin rubber tissue are then laid over the grafted surface, these being first sterilized in a bichloride or carbolic acid solution and then drawn through the salt solution. A mass of sterilized gauze is then placed over this, and finally over all a well applied gauze bandage. From the successful experience he has had in Thiersch's skin-grafting, he feels himself now at perfect liberty to practice a very extensive and thorough removal of diseased tissue, knowing that he can, by this method, cover up the surface without resort to a plastic operation, and without running the risk of leaving behind a large ulcer for many months afterwards.

A very interesting article was read by Dr. D. Bryson Delavan, at a recent meeting of the Section of Laryngology of the Academy of Medicine of this city. The title of the paper was, "The Surgical Treatment of Tubercular Laryngitis," and embodied the results of a visit the author made to the clinic of Professor Krause, at Berlin, last summer. Dr. Delavan related the technique of operation as performed by Prof. Krause. The larynx is first anaesthetized by means of cocoaine. The surface of the ulcer is then thoroughly scraped with a sharp curette, and when bleeding has ceased, lactic acid is applied. Krause insists that the lactic acid must be rubbed in so as to be thoroughly incorporated with the parts under treatment, in order that the destruction of the bacilli be assured. A twenty to fifty per cent. solution of the acid is usually well tolerated and sufficient for the purpose. He considers careful rubbing in of the acid of itself frequently sufficient without any scraping process.

For the success of the operation, it is absolutely necessary that the disease be quiescent. In cases in which no inflammation is present, large masses of œdematous tissue may be removed with the most signal success in the relief of the dyspnœa, cough and dysphagia. Under the influence of the lactic acid treatment, healing is rapid, and the resulting cicatrix presents a remarkably healthy appearance.

There was a large attendance at the meeting, and the paper was ably and intelligently discussed.

Dr. H. Holbrook Curtis, who has a very large experience in the treatment of such affections, said that he had been in the habit of using lactic acid in these cases for the last two years, but did not use the curette of Krause. By this method he had cured two very severe cases of tubercular laryingitis. One of them was the worst he had ever seen, there being perforation, and finally ulceration, externally, through the larynx. This gentleman was now cured, as far as the larynx was concerned. The medicament he found most efficient in combination with lactic acid was the deep inhalation of iodoform and ether. He felt certain that iodoform prevented the bacilli from invading the system, and made the soil less fertile for their development. There was another medicament to which Dr. Curtis called the attention of the section, and that was inhalations of peroxide of hydrogen every two hours. He thought that peroxide of hydrogen had a very beneficial effect upon the bronchial mucous membrane, as well as upon ulcerations of the larynx, and materially assisted in the cure of these cases of tubercular laryngitis. He thought a brilliant era was opening up for the treatment of tubercular laryngitis.

Dr. Gleitzman said that he had published in 1886 a series of experiments he had made on patients with lactic acid, and he was the first to call the attention of the profession to this mode of treatment on this side of the water. He had operated by this method on a patient two years ago, who had a very severe form of tubercular laryngitis, and she had remained well ever since. He did not quite agree with Dr. Delavan as to the strength of the acid to be used, for he did not confine himself to 25 or 50 per cent. solutions, but was in the habit of increasing it to 75, 80, and even 100 per cent. He thought this method would keep on improving, and that they would in time be able to cure a great many patients with tubercular diseases of the larynx who are at present doomed to death.

Dr. Francis Delafield, speaking at a recent clinic held at the College of Physicians and Surgeons on the subject of acute Bright's disease, said that all cases of acute Bright's might be grouped into three classes.

The first class is one in which the principal share in the morbid process is taken by the blood-vessels, and in which the principal evidence of inflammation is matter exuded from the vessels. Such a form of nephritis varies in severity and fatality.

The second class is one in which the principal share in the morbid process is taken by the renal epithelium, and in which the evidences of inflammation are certain changes in these cells. This form is generally secondary to other diseases, and gives no constitutional symptoms.

The third class is that in which the principal share in the morbid process is taken by the stroma, and in which the evidences of inflammation are permanent connective tissue in the stroma, in addition to the lesions of exudative nephritis. This form of nephritis is a permanent and progressive one and attended by well-marked constitutional symptoms.

